# Supplementary material for: Associations of HIV and prevalent type 2 diabetes mellitus in the context of obesity in South Africa
Source: J Multimorb Comorb. 2024 Oct 28;14:26335565241293691. doi: 10.1177/26335565241293691 (PMC11528680; doi:10.1177/26335565241293691)
Supplement: Supplemental Material - Associations of HIV and prevalent type 2 diabetes mellitus in the context of obesity in South Africa [file sj-pdf-1-cob-10.1177_26335565241293691.pdf]

Supplementary Table 1. Parameters for multivariate fractional polynomial models estimating adjusted prevalence, prevalence difference and prevalence ratio for type 2 diabetes mellitus (dichotomous endpoint) by anthropometric categories among adults aged ≥20 years old: South Africa Demographic Health Survey, 2016, unweighted

**Variable (X)**	**Male**			**Female**		
	**Transformation**	**β**	**SE**	**Transformation**	**β**	**SE**
Waist circumference						
HIV status	-	-	-	X	-0.383	0.129
WC, categorical	X	0.976	0.164	X	1.115	0.166
Age	$(X/10)^3$ - 73.146	0.054	0.008	ln(X/10) – 1.484	3.317	0.346
Age	$[(X/10)^3$ x ln((X/10)] - 104.659	-0.024	0.004	$(X/10)^3$ – 86.013	-0.004	0.006
Alcohol use	-	-	-	X	-0.734	0.169
Smoking status	X	-0.351	0.174	-	-	-
Wealth index	X + 0.216	0.017	0.008	-	-	-
Waist-to-height ratio						
HIV status	-	-	-	X	-0.379	0.129
WtHR, categorical	X	1.054	0.200	X	0.965	0.168
Age	$(X/10)^3$ -73.064	0.053	0.009	ln(X/10) – 1.484	3.400	0.345
Age	$[(X/10)^3$ x ln((X/10)] -104.514	-0.024	0.004	$(X/10)^3$ – 85.768	-0.003	0.006
Alcohol use	-	-	-	X	-0.738	0.169
Smoking status	X	-0.334	0.176	-	-	-
Wealth index	X + 0.207	0.024	0.008	-	-	-
Body mass index						
HIV status	-	-	-	X	-0.376	0.131
BMI, categorical	X - 1.396	0.449	0.089	X - 2.126	0.526	0.064
Age	$(X/10)^3$ - 72.492	0.058	0.009	ln(X/10) – 1.485	3.216	0.346
Age	$[(X/10)^3$ x ln((X/10)] - 103.506	-0.027	0.004	$(X/10)^3$ – 86.009	-0.003	0.006
Alcohol use	-	-	-	X	-0.644	0.171
Smoking status	X	-0.299	0.177			
Wealth index	X + 0.159	0.020	0.008	-	-	-

Models were sex-stratified and all initially included age, race, area of residence, household wealth index, smoking and alcohol drinking status, and history of TB drug treatment for TB use as covariates.

Only the covariates that were statistically significant at p-value <0.05 were retained in final models and are represented by "X". For example, HIV status was statistically significant in MFP models for females (p<0.001) but not males (p=0.67), and hence HIV status (represented by "X") was included for females but excluded (represented by "–") for males.

Covariates in MFP models are automatically transformed to improve the scaling of the regression (β) coefficients. For example, age (represented by "X") is divided by 10 (*i.e.*, X/10).

WC = waist circumference; WtHR = waist-to-height ratio; BMI = body mass index; In = natural log transformation.

Supplementary Table 2. Unadjusted prevalence of type 2 diabetes mellitus* (dichotomous endpoint) according to waist circumference, waist-to-height ratio and body mass index categories by HIV serostatus and sex males aged ≥20 years old: unweighted South Africa Demographic Health Survey, 2016

**Adiposity category**	**Unadjusted estimate (95%CI);** ***Males***					
	**Prevalence**		**Prevalence**	**P value**	**Prevalence**	**P value**
	**PWOH**	**PWH**	**difference**[α]		**ratio**[α]	
Overall	10.0 (8.6, 11.3)	9.2 (5.9, 12.5)	-0.8 (-4.4, 2.7)	0.651	0.92 (0.56, 1.27)	0.117
Waist circumference						
Normal	5.1 (3.9, 6.3)	4.4 (2.0, 6.8)	-0.7 (-3.4, 2.0)	0.618	0.86 (0.34, 1.38)	0.635
Elevated	26.8 (22.4, 31.1)	25.5 (13.5, 37.5)	-1.3 (-14.0, 11.5)	0.844	0.95 (0.48, 1.42)	0.847
Waist-to-height ratio						
Normal	3.2 (2.1, 4.3)	3.4 (0.9, 5.9)	0.2 (-2.5, 2.9)	0.896	1.06 (0.20, 1.91)	0.895
Elevated	20.2 (17.2, 23.20	15.5 (8.9, 22.1)	-4.7 (-11.9, 2.6)	0.204	0.77 (0.42, 1.11)	0.251
Body mass index						
Underweight	2.3 (-0.3, 4.9)	5.3 (-1.8, 12.4)	2.9 (-4.6, 10.5)	0.443	2.28 (-1.72, 6.28)	0.358
Normal	5.5 (4.0, 6.9)	4.1 (1.3, 7.0)	-1.3 (-4.5, 1.8)	0.408	0.76 (0.20, 1.31)	0.452
Overweight	15.6 (12.0, 19.3)	12.5 (4.4, 20.6)	-3.1 (-12.0, 57.6)	0.490	0.80 (0.25, 1.35)	0.526
Obese	26.5 (20.8, 32.2)	26.1 (8.1, 44.0)	-0.4 (-0.19.3, 18.4)	0.964	0.98 (0.27, 1.69)	0.964

[α] PWOH are the reference group.

* Diabetes = HbA1c ≥6.5% and/or current use of oral hypoglycemic medicines and/or insulin.

Elevated waist circumference if ≥94cm (for males). Elevated waist-to-height ratio if ≥0.5. Body mass index categories: underweight: <18.5 kg/m^2; normal 18.5-24.9 kg/m^2; overweight 25-29.9 kg/m^2; obese ≥30 kg/m^2.

PWH = people with HIV; PWOH = people without HIV.

Supplementary Table 3. Unadjusted prevalence of type 2 diabetes mellitus* (dichotomous endpoint) according to waist circumference, waist-to-height ratio and body mass index categories by HIV serostatus and sex among females aged ≥20 years old: unweighted South Africa Demographic Health Survey, 2016

**Adiposity category**	**Unadjusted estimate (95%CI);** ***Females***					
	**Prevalence**		**Prevalence**	**P value**	**Prevalence**	**P value**
	**PWOH**	**PWH**	**difference**[α]		**ratio**[α]	
Overall	16.7 (15.3, 18.1)	8.5 (6.7, 10.4)	-8.2 (-10.5, -5.9)	<0.001	0.51 (0.39, 0.63)	<0.001
Waist circumference						
Normal	3.8 (2.4, 5.1)	4.2 (2.0, 6.4)	0.4 (-2.2, 3.1)	0.318	1.11 (0.39, 1.83)	0.746
Elevated	22.1 (20.2, 23.9)	10.3 (7.9, 12.8)	-11.7 (-14.8, -8.7)	<0.001	0.47 (0.36, 0.59)	<0.001
Waist-to-height ratio						
Normal	3.7 (2.2, 5.2)	5.1 (2.6, 7.6)	1.4 (-1.5, 4.30	0.953	1.38 (0.51, 2.25)	0.314
Elevated	21.4 (19.6, 23.3)	9.7 (7.4, 12.1)	-11.7 (-14.7, -8.7)	<0.001	0.45 (0.34, 0.57)	<0.001
Body mass index						
Underweight	3.7 (-1.3, 8.7)	6.7 (-2.3, 15.6)	2.9 (-7.3, 13.2)	0.567	1.80 (-1.64, 5.23)	0.548
Normal	6.4 (4.5, 8.2)	4.8 (2.3, 7.4)	-1.5 (-4.7, 1.7)	0.348	0.76 (0.29, 1.22)	0.377
Overweight	11.9 (9.5, 14.2)	6.9 (3.9, 9.9)	-5.0 (-8.8, -1.2)	0.009	0.58 (0.30, 0.86)	0.025
Obese	26.7 (24.2, 29.3)	11.6 (8.2, 15.1)	-15.1 (-19.4, -10.8)	<0.001	0.44 (0.31, 0.57)	<0.001

[α] PWOH are the reference group.

* Diabetes = HbA1c ≥6.5% and/or current use of oral hypoglycemic medicines and/or insulin.

Elevated waist circumference if ≥80cm (for females). Elevated waist-to-height ratio if ≥0.5. Body mass index categories: underweight: <18.5 kg/m^2; normal 18.5-24.9 kg/m^2; overweight 25-29.9 kg/m^2; obese ≥30 kg/m^2.

PWH = people with HIV; PWOH = people without HIV.

Supplementary Figure 1. Covariate balance before and after propensity score matching of (a) men and (b) women (≥20 years old) by HIV serostatus for sensitivity analysis: unweighted South Africa Demographic Health Survey, 2016

(a) (b)

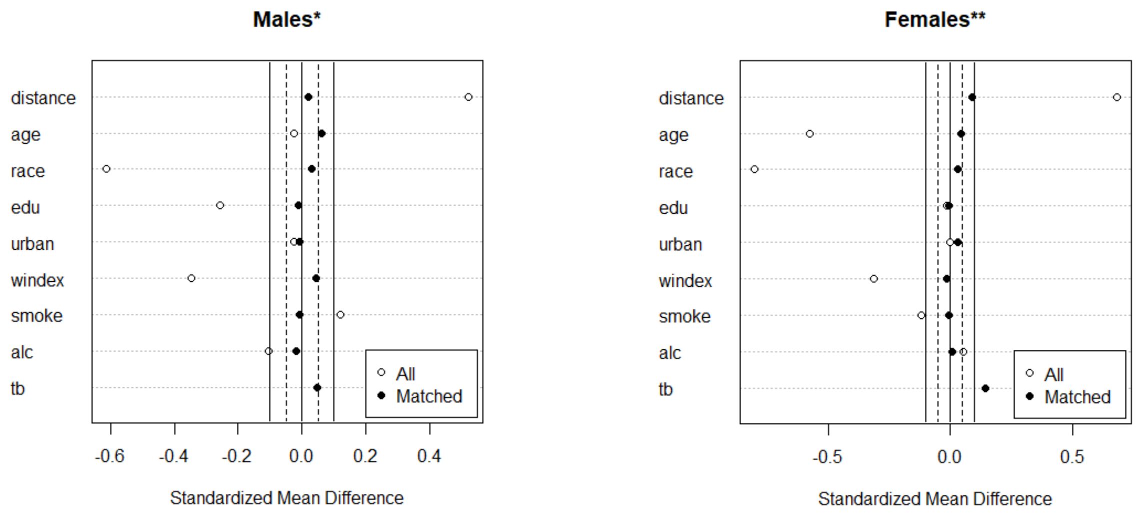

* Among men, there were 1,634 PWOH and 312 PWH before matching; and 312 PWOH and 312 PWH after matching.

** Among women, there were 2,584 PWOH and 901 PWH before matching; and 901 PWOH and 901 PWH after matching.

PWH = people with HIV; PWOH = people without HIV.

Supplementary Figure 2. Mean glycated (HbA1c) (continuous endpoint) according to waist circumference, body mass index and waist-to-height ratio by HIV serostatus and sex among adults aged ≥20 years old: unweighted South Africa Demographic Health Survey, 2016

(a)

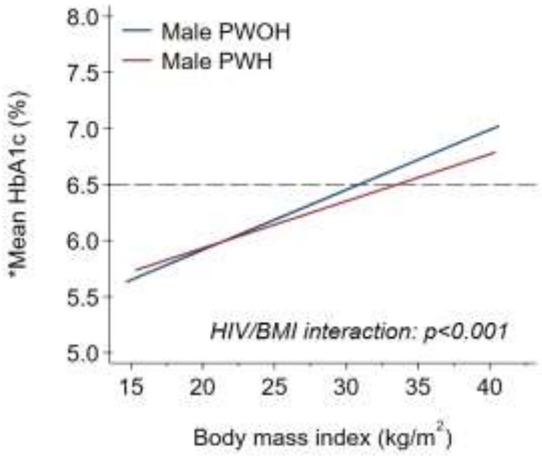

(b)

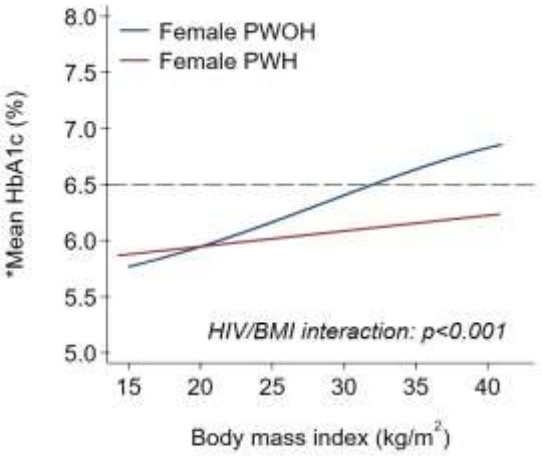

(c)

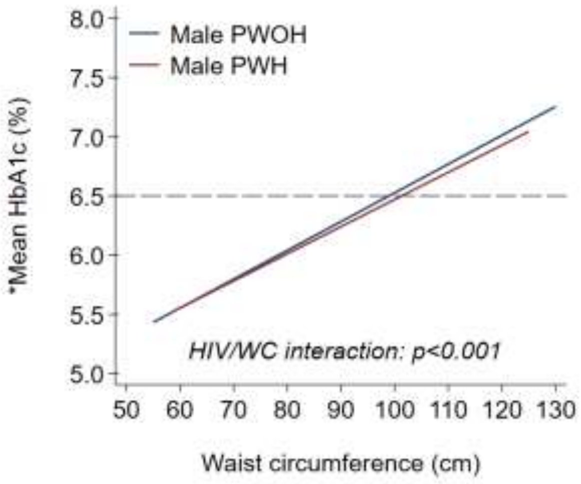

(d)

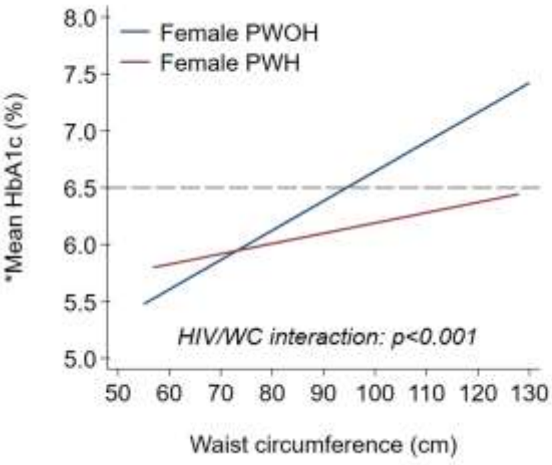

PWH = people with HIV; PWOH = people without HIV.

(e)

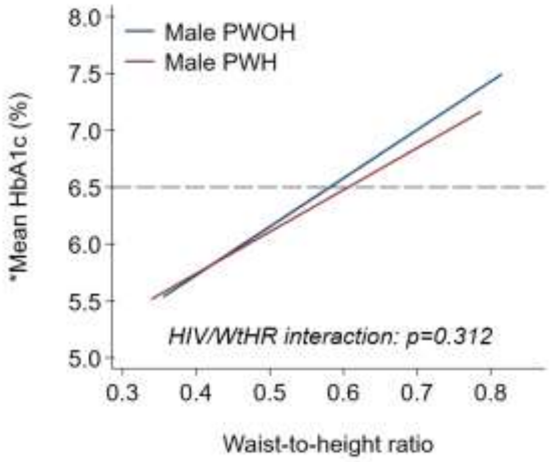

(f)

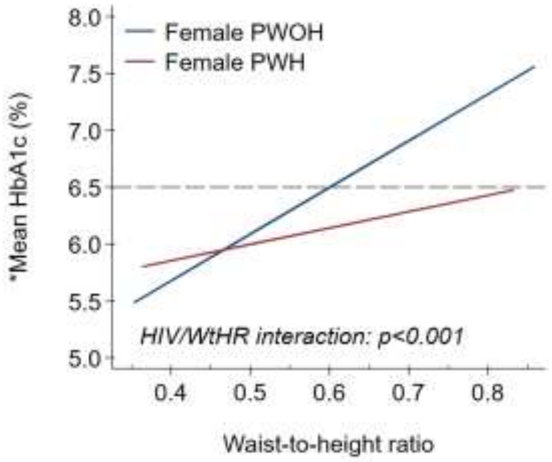

***** Estimated from univariable fractional polynomial model of specific anthropometric index without adjustment.

PWH = people with HIV; PWOH = people without HIV.
